# “When should primary angiitis of the central nervous system (PACNS) be suspected?”: literature review and proposal of a preliminary screening algorithm

**DOI:** 10.1007/s10072-020-04583-3

**Published:** 2020-08-10

**Authors:** Cristina Sarti, Antonella Picchioni, Roberta Telese, Marco Pasi, Ylenia Failli, Giovanni Pracucci, Daniele Cammelli, Domenico Inzitari

**Affiliations:** 1 NEUROFARBA Department, Neuroscience Section, University of Florence, Largo Brambilla 3, 50134 Florence, Italy; 2 Department of Neurosciences, Imaging and Clinical Sciences, University of Chieti-Pescara, Chieti, Italy; 3 Department of Neurology, Université Lille, CHU Lille, Lille, France; 4 Department of Experimental and Clinical Medicine, University of Florence, Largo Brambilla 3, 50134 Florence, Italy

**Keywords:** PACNS, CNS vasculitis, Magnetic resonance imaging, Cerebral angiography, Primary angiitis of the CNS

## Abstract

**Background:**

Primary angiitis of the CNS (PACNS) is a process causing variously combined neurological disturbances. Its rarity and kaleidoscopic presentation make it difficult to diagnose and even to suspect.

**Objective:**

(1) To provide an up-to-date review on PACNS and (2) to create a preliminary screening algorithm based on clinical and radiological first-level data, useful to suspect PACNS and guide further investigations.

**Methods:**

Review of PUBMED case series on PACNS, published from 2002 to 2017, collection of frequencies of clinical and neuroimaging features and calculation of median values. Classification of features as “major” or “minor” if frequency was higher or lower than median value. Combination of features in sets of criteria represented by all possible combinations of major and minor clinical and neuroradiological features. Application of criteria to published PACNS case reports and selection of the ones best identifying patients with definite PACNS.

**Results:**

We reviewed 24 case series. “Major” clinical features were headache, stroke, cognitive impairment, focal neurological deficits; “minor” were seizures, altered consciousness, psychiatric disorders. “Major” neuroradiological features were multiple parenchymal lesions, parenchymal/meningeal contrast enhancement, magnetic resonance angiography vessel abnormalities, vessel wall enhancement; “minor” were parenchymal/subarachnoid hemorrhage, single parenchymal lesion. The selected sets of criteria able to identify all PACNS patients were (1) one clinical (major/minor) + one major neuroradiological feature; and (2) Two clinical (≥ 1 major) + one minor neuroradiological feature.

**Conclusion:**

Our review provides a detailed clinical/neuroradiological picture of PACNS. The proposed algorithm should be regarded as a preliminary screening tool to move the first steps towards PACNS diagnosis that needs validation.

## Introduction

Primary angiitis of the central nervous system (PACNS) is a rare form of vasculitis affecting only CNS blood vessels, with no evidence of vasculitis in other organs and systems [1]. Recognized for the first time in the 1950s [1], it has been poorly described until 1988, when Calabrese and Mallek proposed their diagnostic criteria [2], requiring the presence of an otherwise unexplained neurological or psychiatric deficit, the presence of either classic angiographic or histopathological features of angiitis of the CNS, and no evidence of systemic vasculitis or any other disorder that could cause or mimic the angiographic or pathological features of the disease.

The first large case series dates back to 2007, when Salvarani et al. published a retrospective study on 101 patients [3]. Even though in the last decade attention to this nosological entity has risen, it still remains a rare and poorly described disease.

Patients with PACNS can present with non-specific neurological disorders and symptoms, such as ischemic or hemorrhagic stroke, cognitive impairment, headache, seizures, and psychiatric disorders [1]. If untreated, PACNS can progress, leading to moderate-severe disability or even death, while, if recognized and treated early, complete recovery may occur. For this reason, early diagnosis and treatment are mandatory [4].

The rarity of this disease, its non-specific clinical presentation, and the lack of validated diagnostic tests, however, make it difficult both to diagnose and suspect [1, 4].

The aims of this study are (1) to provide a detailed review of the literature on this subject and (2) to create a preliminary screening algorithm to suspect PACNS, based on neurological clinical features and non-invasive neuroradiological signs. This could subsequently help in the selection of patients who deserve further and more invasive investigations such as cerebral angiography, lumbar puncture, and cerebral biopsy.

## Methods

### Phase A: case series narrative review

PubMed case series published between 2002 and 2019 were reviewed in order to examine patients’ clinical (neurological) and neuroradiological characteristics. Search terms were “primary angiitis of the CNS,” “PACNS,” and “CNS vasculitis”. All case series published in English and reporting at least 3 adult patients with a diagnosis of PACNS were included. The frequencies of neurological and non-invasive neuroradiological features reported on each article were collected. The overall frequency of each feature, derived from pooled data of all studies, was calculated.

### Phase B: classification of clinical and neuroradiological features as “major” or “minor”

Median frequency value of clinical and neuroradiological features was calculated. Major features were the ones whose frequency was higher or equal to the median value, while minor features were the ones whose frequency was lower than the median value.

### Phase C: combination of features in sets of screening criteria

All possible combinations of major and minor clinical and neuroradiological features were identified.

### Phase D: elaboration of the screening algorithm

All literature case reports of adult patients with an already definite diagnosis of PACNS, according to the Calabrese and Mallek [2] criteria, written in English and published from 2011 to 2017, were searched for on PubMed. The sets of criteria elaborated in Phase C were applied to these case reports, and the ones identifying patients affected by PACNS were chosen as PACNS preliminary screening criteria.

## Results

### Phase A: case series review

We found 24 case series [1, 3, 5–28], amounting to a total of 585 patients with a diagnosis of PACNS, 41% biopsy proven. No duplications of patients were included. The overall frequency of clinical and neuroradiological features is shown in Table 1. Tables 2, 3, 4, and 5 display the clinical and neuroradiological features of each case series in detail.

**Table 1:** Summary of clinical and neuroradiological data

CLINICAL FEATURES (*n* = 585)	
Headache	320/560 (57.1%)
Cognitive impairment	190/445 (42.7%)
Stroke/TIA	96/216 (44.4%)
Focal neurological deficits	≥ 340/564 (≥ 60.3%)
Seizure	144/536 (26.9%)
Impaired level of consciousness	67/219 (30.6%)
Psychiatric/mood disorders	31/148 (20.9%)
MRI (*n* = 558 patients)	
Multiple lesions	228/334 (68.3%)
Single lesion	26/222 (11.7%)
Infarction	280/445 (62.9%)
Intraparenchymal or subarachnoid hemorrhage	78/380 (20.5%)
ENHANCED MRI (*n* = 425 patients)	
Parenchymal or meningeal contrast enhancement	198/425 (46.6%)
Black blood MRI (*n* = 20 patients)	
Vessel wall contrast enhancement	17/20 (85%)
MRA (*n* = 352 patients)	
Vessel abnormalities	240/352 (68.2%)

**Table 2:** Clinical findings

	Hajj-Ali et al., 2002 [1]	Volcy et al., 2004 [5]	McLaren et al., 2005 [6]	Kuker et al., 2008 [7]	Molloy et al., 2008 [8]	Lee et al., 2009 [9]	Pizzanelli et al., 2011 [10]	Kreamer et al., 2011 [11]	Neel et al., 2012 [12]	Pfefferkorn et al., 2012 [13]	Pourmahmoodian et al., 2012 [14]	Coronel-Restrepo et al., 2013 [15]	Cosottini et al., 2014 [16]	Geri et al., 2014 [17]	Oon et al., 2014 [18]	Vera-Lastra et al., 2014 [19]	Salvarani et al., 2015 [20]	Singhal et al., 2016 [21]	Becker et al., 2017 [22]	De Boysson et al., 2017 [23]	Niu et al., 2017 [25]	Schuster et al., 2017 [26]	Peng et al., 2017 [27]	Harsha et al., 2017 [28]
	(*n* = 16)	(n = 5)	(*n* = 12)	(*n* = 16)	(*n* = 38)	(n = 4)	(*n* = 8)	(*n* = 21)	(*n* = 8)	(*n* = 4)	(*n* = 3)	(*n* = 3)	(*n* = 8)	(*n* = 18)	(*n* = 12)	(*n* = 12)	(*n* = 163)	(*n* = 47)	(*n* = 25)	(*n* = 102)	(*n* = 19)	(*n* = 31)	(*n* = 5)	(*n* = 5)
Headache	88% (14)	80% (4)	33.3% (4)	ND	74% (28)	ND	75% (6)	42.9% (9)	37% (3)	100% (4)	33.3% (1)	66.7% (2)	62.5% (5)	22.2% (4)	91.66% (11)	66% (8)	59.5% (97)	49% (23)	36% (9)	51% (52)	73.7% (14)	64.5% (20)	40% (2)	ND
Cognitive impairment	44% (7)	ND	8.3% (1)	ND	ND	ND	ND	38.1% (8)	ND	ND	Confusion 33% (1)	66.7% (2)	Acute confusional state 12.5% (1)	22.2% (4)	41.66% (5)	50% (6)	54% (88)	ND	28% (7)	42% (43)	31.6% (6)	35.5% (11)	ND	ND
Stroke/TIA	43% (7)	ND	ND	Stroke 81.2% (13), TIA 18.7% (3)	ND	ND	ND	47.6% (10)	ND	ND	ND	ND	ND	ND	ND	ND	Persistent neurologic deficit or stroke 40.5% (66)	ND	ND	ND	ND	ND	ND	ND
Focal neurological deficits	63% [10]	≥ 80% [4]	≥ 33.3% [4]	ND	64% [24]	75% [3]	100% [8]	≥ 61.9% [13]	100% [8]	≥ 75% [3]	100% [3]	100% [3]	62.5% [5]	≥ 44.4% [8]	50% [6]	83% [10]	40.5% (66)	60% [28]	≥ 52% [13]	79% (81)	≥47.4% [9]	83.9% [26]	100% [5]	ND
	Visual disturbance (diplopia) 14% [2]; ataxia 14% [2]; speech abnormalities 7% [1]	Motor paresis 80% [4], aphasia 20% [1]	Motor paresis 33.3% [4], aphasia 25% [3], cerebellar signs 16% [2], lateral medullary syndrome 8.3% [1], Anton- Babinski syndrome 8.3% [1]			Motor paresis 50% [2], visual disturbance (field deficit) 25% [1], aphasia 25% [1]	Motor paresis 75% [6], sensory disturbances 25% [2], visual disturbance (field deficit) 25% [2]	Motor paresis 61.9% [13], ataxia 28.6% [6], aphasia 23.8% [5], dysarthria 19% [4], vertigo 9.5% [2]		Motor paresis 75% [3], speech disturbances 75% [3], sensory disturbances 50% [2],	Motor paresis 100% [3], aphasia 33% [1], dysarthria 33% [1], ataxia 33% [1]	Motor paresis 100% [3], aphasia 100% [3]	Motor paresis 37.5% [3], dysarthria 12.5% [1], sensory disturbances 12.5% [1]	Motor paresis 44.4% [8], aphasia 22.2% [4], ataxia 22.2% [4], cranial nerve involvement 11.1% [2], sensory disturbances 5.6% [1], vertigo 11.1% [2], visual field defect 5.6% [1])		Motor paresis 41.7% [5], speech disturbances 25% [3], sensory disturbances (numbness, tingling) 33% [4], Ataxia 33% [4],	Persistent neurologic deficit or stroke 40.5% (66)		Motor paresis 52% [13],sSpeech Disorders 32% [8], sensory disturbances 44% [11], cranial Nerve Deficits 24% [6], ataxia 20% [5], vertigo 12% [3],		Motor paresis 47.4% [9]		Dizziness/ver tigo 100% [5], sensory disturbances (facial numbness) 40% [2], dysarthria 20% [1]	
Seizure	21% [3]	60% [3]	41.6% [5]	ND	47% [18]	25% [1]	ND	9.5% [2]	25% [2]	ND	66.6% [2]	ND	ND	22.2% [4]	8.3% [1]	17% [2]	20.2% [33]	28% [13]	24% [6]	35% [36]	26.3% [5]	25.8% [8]	ND	ND
Impaired level of consciousness	ND	40% [2]	ND	ND	Diffuse neurological deficit 50% [19]	ND	37.5% [3]	ND	ND	ND	ND	33% [1]	37.5% [3]	11.1% [2]	ND	50% [6]	ND	ND	16% [4]	26% [27]	ND	ND	ND	ND
Psychiatric/mood disorders	ND	ND	ND	ND	ND	ND	ND	ND	ND	ND	33% [1]	ND	ND	11.1% [2]	ND	ND	ND	ND	24% [6]	22% [22]	ND	ND	ND	ND
Other findings	Blurred vision 14% [2]	Visual disturbance (scotomata) 20% [1]	Encephalitic syndrome 16.6% [2]					Visual disturbance 33.3% [7]		Visual disturbance (blurred vision) 25% [1]	Encephalopathy 33% [1]			Decreased visual acuity 5.6% [1],		Visual disturbances 41.6% [5]			Visual Disturbances 12% [3]					

*ND no data

**Table 3:** MRI features

	Hajj-Ali et al., 2002 [1]	Volcy et al., 2004 [5]	McLaren et al., 2005 [6]	Kuker et al., 2008 [7]	Molloy et al., 2008 [8]	Lee et al., 2009 [9]	Pizzanelli et al., 2011 [10]	Kreamer et al., 2011 [11]	Neel et al., 2012 [12]	Pfefferkorn et al., 2012 [13]	Pourmahmoodian et al., 2012 [14]	Coronel-Restrepo et al., 2013 [15]	Cosottini et al., 2014 [16]	Geri et al., 2014 [17]	Oon et al., 2014 [18]	Vera-Lastra et al., 2014 [19]	Salvarani et al., 2015 [20]	Singhal et al., 2016 [21]	Becker et al., 2017 [22]	De Boysson et al., 2017 [23]	Niu et al., 2017 [25]	Schuster et al., 2017 [26]	Peng et al., 2017 [27]	Harsha et al., 2017 [28]
	(*n* = 16)	(*n* = 5)	(*n* = 12)	(*n* = 16)	(*n* = 38)	(*n* = 4)	(*n* = 8)	(*n* = 21)	(*n* = 8)	(*n* = 4)	(*n* = 3)	(*n* = 3)	(*n* = 8)	(*n* = 18)	(*n* = 12)	(*n* = 12)	(*n* = 163)	(*n* = 47)	(*n* = 25)	(*n* = 102)	(*n* = 19)	(*n* = 31)	(*n* = 5)	(*n* = 5)
Performed in	81.25% (13)	100% (5)	100% (12)	100% (16)	[79% [30]]	100% [4]	100% [8]	100% [21]	100% [8]	100% [4]	[66% [2]]	100% [3]	100% [8]	100% [18]	100% [12]	100% [12]	91.4% (149)	100% [47]	96% [24]	100% (102)	100% [19]	100% [31]	100% [5]	100% [5]
[Enhanced in]				[100% [16]]	79% [30]	[75% [3]]		[100% [21]]	[100% [8]]		66% [2]	[33% [1]]			[100% [12]]		[91.4% (149)]		[96% [24]]	[83% (85)]	[100% [19]]	[100% [31]]	[100% [5]]	[100% [5]]
Abnormal in	77% [10]	ND	100% [12]	ND	100% [30]	100% [4]	100% [8]	100% [21]	100% [8]	100% [4]	100% [2]	100% [3]	ND	100% [18]	100% [12]	ND	ND	100% [47]	100% [24]	ND	100% [19]	100% [31]	100% [5]	100% [5]
Infarction	30.7% [4]	ND	75% [9]	62.5% [10]	ND	ND	50% [4]	76.2% [16]	87.5% [7]	100% [4]	50% [1]	ND	25% [2]	ND	91.6% [11]	33.3% [4]	54.4% (81)	81% [33]	ND	73% (74)	ND	54.8% [17]	ND	ND
Multiple lesions	23% [3]	60% [3]	75% [9]	93,7% [15]	ND	50% [2]	50% [4]	100% [21]	ND	ND	100% [2]	66.7% [2]	62.5% [5]	100% [18]	ND	67% [8]	45.6% (41/90)^3^	66% [31]	ND	94.1% (48/51) ^24^	63% [12]	96,7% [30]	ND	80% [4]
Single lesion	ND	40% [2]	25% [3]	ND	ND	50% [2]	ND	0% (0)	ND	ND	0% (0)	33,3% [1]	ND	0% (0)	8.4% [1]	ND	7.8% (7/90) ^3^	ND	ND	5.9% (3/51)^24^	37% [7]	ND	ND	0% (0)
Parenchymal/meningeal contrast enhancement (PCE or MCE when specified)	ND	ND	ND	6.5% [1]	50% [15]	100% (3/3)	ND	38% [8]	25% [2]	ND	100% [2]	100% [1]	ND	ND	MCE 33% [4]	ND	40.3% [59]	ND	67% [16]	47% (40/85), PCE 41% (35/85), MCE28% (24/85)	100% [19]	54.8% [17]	PCE 100% [5], MCE40% [2]	100% [5]
Intracranial (ICH)/subarachnoid (SAH)/not specified (H) hemorrhage	ICH 15,3% [2], SAH 7,7% [1]	ND	ND	ND	H 16,7% [5]	ND	ICH or SAH 62.5% [5]	ICH 4.8% [1]	ND	ND	50% [1]	33.3% [1]	ICH 12.5% [1] SAH 37.5% [3]	21,1% [4]	ND	ND	7.8% (7/90) ^3^	ICH 9% [4]; SAH 2% [1]	ICH 16.7% [4]	H 33% [34]	ND	16.1% [5]	ND	ND
Mass-like lesion	ND	ND	ND	ND	100% [30]	100% [4]	ND	ND	ND	ND	ND	ND	ND	ND	ND	ND	ND	ND	29.2% [7]	12% [12]	ND	ND	ND	ND
Vessel wall contrast enhancement (black blood MRI)	ND	ND	ND	81,25% [13]	ND	ND	ND	ND	ND	100% [4]	ND	ND	ND	ND	ND	ND	ND	ND	ND	ND	ND	ND	ND	ND

*ND no data

**Table 4:** MRA features

	Hajj-Ali et al., 2002 [1]	Volcy et al., 2004 [5]	McLaren et al., 2005 [6]	Kuker et al., 2008 [7]	Molloy et al., 2008 [8]	Lee et al., 2009 [9]	Pizzanelli et al., 2011 [10]	Kreamer et al., 2011 [11]	Neel et al., 2012 [12]	Pfefferkorn et al., 2012 [13]	Pourmahmoodian et al., 2012 [14]	Coronel-Restrepo et al., 2013 [15]	Cosottini et al., 2014 [16]	Geri et al., 2014 [17]	Oon et al., 2014 [18]	Vera-Lastra et al., 2014 [19]	Salvarani et al., 2015 [20]	Singhal et al., 2016 [21]	Becker et al., 2017 [22]	De Boysson et al., 2017 [23]	Niu et al., 2017 [25]	Schuster et al., 2017 [26]	Peng et al., 2017 [27]	Harsha et al., 2017 [28]
	(*n* = 16)	(*n* = 5)	(*n* = 12)	(*n* = 16)	(*n* = 38)	(*n* = 4)	(*n* = 8)	(*n* = 21)	(n = 8)	(*n* = 4)	(*n* = 3)	(*n* = 3)	(*n* = 8)	(*n* = 18)	(*n* = 12)	(*n* = 12)	(*n* = 163)	(*n* = 47)	(*n* = 25)	(*n* = 102)	(*n* = 19)	(*n* = 31)	(*n* = 5)	(*n* = 5)
Performed in	NP*	NP*	NP*	100% (16)	NP*	100% (4)	NP*	81% (17)	83% (7)	100% (4)	33% (1)	33% (1)	100% (8)	39% (7)	100% (12)	NP*	79% (129)	32% (15)	MRA or CT angiography performed in 72% (18)	89.2% (91)	NP*	100% (31)	20% (1)	100% (5)
Abnormal in				93,5% (15)		25% [1]		47,1% [8]	Abnormal at onset 25% [2] Abnormal at follow-up 50% [4]	100% [4]	100% [1]	100% [1]	87,5% [7]	71,4% [5]	66% [8]		≥90,4% (103)	NO-specific data	61% [11]	62.6% [56]		51,6% [16]	0% (0)	0% (0)
Other findings														Proximal lesions 85,7% [6]; distal lesions 57,1% [4]			Large/proximal vessel vasculitis 65.8% (75); small/distal vessel vasculitis 90.4% (103)							

*NP not performed

**Table 5:** Application of criteria to literature case reports

	Major clinical criteria				Minor clinical criteria			Major neuroradiological criteria				Minor neuroradiological criteria			
	Headache	Cognitive impairment	Stroke/TIA	Focal neurological deficit	Seizure (s)	Impaired level of consciousness	Psychiatric alterations	Multiple MRI lesions	Parenchymal or meningeal contrast enhancement	MRA abnormalities	Vessel wall contrast enhancement on black blood MRI	Single MRI lesion	Intraparenchymal or subarachnoid hemorrhage	Clinical + neuroradiological criteria*	Combination (s)
Al Share, 2017 [29]	x								x					C:1 > + N:1 >	A
Kumar, 2017 [30]		x							x			x		C:1 > + N:1 >and 1 <	A
Takahashi, 2017 [31]	x				x			x	x					C:1 > and 1 < + N:2 >	A
Lee, 2017 [32]	x			x					x			x		C: 2 > + N:1 > and 1 <	A & B
Arif, 2017 [33]	x				x			x						C:1 > and 1 < + N:1 >	A
Mizuno, 2016 [34]				x				x	x					C:1 > + N:2 >	A
Sun, 2016 [35]	x			x	x							x		C: 2 > and 1 < + N:1 <	B
Benson, 2016 [36]	x			x					x					C:2 > + N:1 >	A
Pillai, 2016 [37]			x	x				x						C:2 > + N:1 >	A
Fang, 2015 [38]		x				x		x	x				x	C:1 > and 1 < + N:2 > and 1 <	A & B
Kim, 2015 [39]						x	X	x	x					C:2 < + N:2 >	A
Bajaj, 2015 [40]	x				x			x	x					C:1 > and 1 < + N:2 >	A
Kim SI, 2015 [41]					x				x			x		C:1 < + N:1 > and 1 <	A
Killeen, 2015 [42]	x	x		x					x			x		C:3 > + N:1 > and 1 <	A & B
Gan, 2015 [43]	x	x							x			x		C:2 > + N:1 > and 1 <	A & B
Huang, 2015 [44]	x	x						x	x					C:2 > + N:2 >	A
Okunomiya, 2014 [45]		x						x	x					C:1 > + N:2 >	A
Safouris, 2014 [46]			x	x				x						C:2 > + N:1 >	A
Michiels, 2014 [47]		x		x	x			x					x	C:2 > and 1 < + N:1 > and 1 <	A & B
Gaillard, 2014 [48]				x				x	x					C:1 > + N:2 >	A
Rao, 2014 [49]	x							x	x					C:1 > + N:2 >	A
Noh, 2014 [50]			x	x				x		x	x			C:2 > + N:3 >	A
Safouris, 2013 [46]			x	x				x						C:2 > + N:1 >	A
Orr, 2013 [51]	x			x				x	x					C:2 > + N:2 >	A
Lyra, 2013 [52]				x				x	x				x	C:1 > + N:2 > and 1 <	A
Rosenberg, 2013 [53]	x							x	x					C:1 > + N: 2 >	A
Okeda, 2012 [54]					x			x	x					C:1 < + N:2 >	A
Yu, 2011 [55]		x		x	x			x						C:2 > and 1 < + N:1 >	A
Ghavanini, 2011 [56]		x			x				x				x	C:1 > and 1 < + N:1 > and 1 <	A & B
Ho, 2011 [57]				x	x			x	x					C:1 > and 1 < + N:2 >	A
Tanei, 2011 [58]				x				x	x					C:1 > + N:2 >	A
Pires, 2011 [59]		x						x						C:1 > + N:1 >	A

*C number of major (>) or minor (<) clinical criteria present; *N* = number of major (>) or minor (<) neuroradiological criteria present

Combination ‘A’: One clinical (major or minor) + one major neuroradiological feature

Combination ‘B’: Two clinical (at least one major) + one minor neuroradiological feature

#### Neurological clinical features (Tables 1 and 2)

Headache was present in 57.1% (320/560) of patients, cognitive impairment in 42.7% (190/445), stroke or transient ischemic attack (TIA) in 44.4% (96/216), and a focal neurological deficit (such as hemiparesis, aphasia, ataxia, or visual symptoms) in at least 60.3% of patients (≥ 340/564). Moreover, seizures were reported in 26.9% (144/536), confusion or impaired level of consciousness in 30.6% (67/219), and psychiatric or mood disorders in 20.9% (31/148) of patients.

#### MRI (Tables 1 and 3)

MRI was performed in 558 patients and showed multiple, usually not otherwise specified, parenchymal lesions in 68.3% (228/334) of patients and single parenchymal lesion in 11.7% (26/222). Intraparenchymal or subarachnoid hemorrhage was observed in 16.6% (78/380) of patients.

In two cases (Salvarani et al., 2015 [20] and De Boysson et al., 2017 [23]), MRI was performed, but the frequency of specific features was reported only in previous papers based on the same population (Salvarani et al., 2007 [3] and De Boysson et al., 2014 [24]); we referred to those papers to calculate their frequency.

Data concerning contrast enhancement were given for 425 patients: 198 (46.6%) showed parenchymal or meningeal contrast enhancement.

#### Black blood MRI (Tables 1 and 3)

Only two studies (Kuker et al. [7] and Pfefferkorn et al. [13]) reported data on this new MRI technique. Vessel wall contrast enhancement was found in 85% (17/20) of patients.

#### MRA (Tables 1 and 4)

MRA was performed in 346 patients and showed vessel abnormalities (such as single or multiple stenoses, occlusion) in 68.2% (240/352) of patients.

### Phase B: classification of features as major and minor

1. Median of frequencies for clinical features: 42.7%
2. Median of frequencies for neuroradiological features: 46.6%
3. Classification of features in “major” and” minor” (frequencies shown in Table 1):
  *Major clinical features* (≥ 42.7%): Headache, stroke, cognitive impairment, and focal neurological deficits
  *Minor clinical features* (< 42.7%): Seizure(s), altered level of consciousness, and psychiatric disorders
  *Major neuroradiological features* (≥ 46.6%): Multiple parenchymal lesions, parenchymal or meningeal contrast enhancement, vessel abnormalities (single or multiple stenoses/occlusion), and vessel wall contrast enhancement
  *Minor neuroradiological features* (< 46.6%): Parenchymal or subarachnoid hemorrhage and single parenchymal lesion

### Phase C: combination of features in sets of criteria

All the possible combinations of these features were:

A. One clinical (major or minor) + one major neuroradiological feature
B. Two clinical (at least one major) + one minor neuroradiological feature
C. One major clinical + two minor neuroradiological features
D. One major clinical + one minor neuroradiological feature
E. Two major clinical + one minor neuroradiological feature
F. One minor clinical + two minor neuroradiological features
G. One minor clinical + one minor neuroradiological feature

### Phase D: elaboration of the screening algorithm

Thirty-two case reports of adult patients with a definite diagnosis of PACNS, according to the Calabrese and Mallek criteria [2] (29 biopsy proven), published in English from 2011 to 2017, were found on PubMed. The application of the previously mentioned sets of criteria to the 32 case reports is shown on Table 5. The first two combinations (A and B) were found to be verified in all patients with PACNS.

**Fig. 1:**
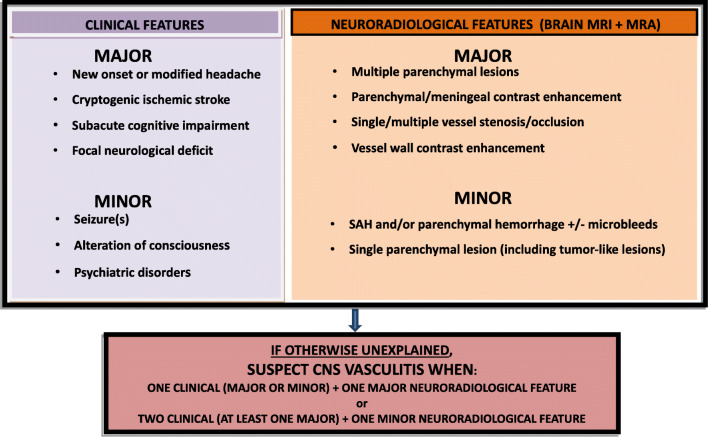
Clinico-radiological screening algorithm

A screening algorithm is then developed (Fig. 1). According to it, PACNS should be preliminary suspected if patients have: (1) one clinical feature (major or minor) associated with one major neuroradiological feature or (2) two clinical features (at least one major) associated with one minor neuroradiological feature. No better explanation for the presenting complaint should be found.

## Discussion

PACNS suspicion and diagnosis are challenging for the low specificity of both neurological and neuroimaging signs, its kaleidoscopic presentation, and the poor knowledge of the disease. Even though the disease is severe and potentially fatal, early recognition and treatment sensibly change its natural history, reducing morbidity and mortality [20].

To the best of our knowledge, this is the first study that provides a detailed review of all literature available on PACNS and the first to propose an operational tool to be used in clinical practice to answer the question: “When should I suspect PACNS?”

Our revision confirms the heterogeneous and non-specific clinical presentation of PACNS. Some symptoms are more frequent, like headache, altered cognition, and persistent neurologic deficits [60], while others are probably underreported, such as anxiety, depression, mood changes, and insomnia [61]. One feature which leads to suspect PACNS is the temporal evolution. Some patients may present with clinical worsening, and others may present within a short-time interval with new multiple acute lesions on brain MRI.

Considering that headache is a widespread complaint in the general population and that it is associated with many neurological disorders, it would be of help to be able to better define the characteristics of headache that could lead to suspect vasculitis. In PACNS case series, no detailed description is given. In PACNS case reports, no univocal nor specific pattern is reported; headache is often described as subacute or chronic with insidious onset, dull, diffuse, sometimes intermittent, worsening with time, sometimes migraine-like, and with a wide range of severity usually of mild-to-moderate intensity. In patients already suffering of headache, it is described as changed from that usually experienced. In the tumor-like form of PACNS, headache can be acute, violent, and associated with vomiting. Headache of the thunderclap variety is extremely rare in PACNS, thus helping in distinguishing it from other neurological conditions that can mimic its clinical picture, such as subarachnoid hemorrhage or reversible cerebral vasoconstriction syndrome. It lacks however clear-cut characteristics [62] and should point to vasculitis; when it is associated with the imaging markers, we have selected based on our review and proposed with our algorithm.

Unlike systemic vasculitis, fever, night sweats, weight loss, and other constitutional symptoms are uncommon in PACNS (Salvarani, 2015).

Since there are no serologic tests with even moderate sensitivity or specificity for PACNS, serologic tests are mainly useful to rule out alternative diagnoses, such as infection, other systemic autoimmune diseases, or malignancy. Erythrocyte sedimentation rate and C-reactive protein are typically normal in PACNS [3, 20]; thus, elevated inflammatory marker levels should heighten suspicion for a diagnosis other than PACNS [63]. When prompted by the clinical picture, other important tests to rule out secondary causes of CNS vasculitis include [60, 63, 64]:

- Autoantibody panels for systemic vasculitis and autoimmune systemic diseases (antinuclear antibodies, anti-extractable nuclear antigens antibodies, antineutrophil cytoplasmic antibodies, antiphospholipid antibodies, rheumatoid factor).
- Serological tests for infectious causes of systemic vasculitis (these should include serology for varicella-zoster virus, mycobacteria, syphilis, human immunodeficiency virus, and fungi).
- Lumbar puncture (in PACNS cerebrospinal fluid findings are non-specific; common findings include mild-to-moderate lymphocytic pleocytosis and/or elevated protein with normal glucose; still a lumbar puncture may be useful in ruling out VZV-associated CNS vasculitis and other infectious etiologies and malignancy).

Some limitations have to be addressed, related both to the quality of the studies we reviewed and to the methodology we used in creating the algorithm.

Regarding the studies reviewed, case series published until today show a number of critical points:

1. Most studies are retrospective and may be subjected to recall bias.
2. Many studies report that multiple neurological symptoms are often coexisting, but they never give a definite and univocal picture of PACNS. For example, which symptoms are the most frequent and which are the earliest to appear is not specified.
3. Many studies include “focal neurological deficits” as part of the clinical picture of PACNS, but no data are given as to their type of onset nor to their evolution in time.
4. Data concerning imaging techniques are not uniform since in older studies some of them were not available.

Some studies have shown that black blood imaging can non-invasively visualize vessel wall thickening and enhancement patterns helping differentiate vasculitis from other causes of vasculopathy, such as atherosclerosis. In an atherosclerotic lesion, vessel wall thickening and enhancement are usually eccentric, while in vasculitis the wall thickening and enhancement are usually concentric, homogenous, and in a long portion of the vessel. However, the presence of enhancement of vessel wall as a clue to vasculitis is a relatively newly acquired knowledge, and although promising, the number of cases studied with such technique up to date is extremely low, and their diagnostic validity remains unproven. We categorized it as a “major neuroradiological feature”, but further studies more closely estimating its predictive value are necessary. Similarly, 3T MRI, a promising non-invasive technique that could increase intracranial stenosis and vessel wall thickening visualization, has been introduced only in 2010, and by now it is still not available in most clinical centers around the world.

5. The nature of the parenchymal lesions seen on MRI is often not specified in case series. They might be related to white matter lesions, acute or chronic infarcts, and chronic bleedings.

All these factors could have influenced the estimation of the frequency of single features used to elaborate our algorithm.

Regarding the methodology of our study, the following concerns emerged:

1. The total percentage of “focal symptoms” we provide might be underestimated, since, as stated above, some studies only reported the frequency of specific deficits, but not their combination in each patient. For those studies, we decided to consider the deficit with the highest percentage, aware of the fact that patients with isolated different deficits might be lost.
2. Our literature review highlighted that some symptoms and some neuroradiological signs recurred in most of the studies, suggesting that they should be weighted more than others when suspecting PACNS. This led to the idea of categorizing clinical and neuroradiological features as “major” or “minor.” The subsequent step was to combine clinical and radiological signs to provide “screening criteria,” whose application to case reports with a definite diagnosis of PACNS led to select two combinations capable of identifying each of them. The two selected combinations were (A) one clinical feature that could be major or minor associated to one major neuroradiological feature and (B) two clinical features (at least one major) associated to one minor neuroradiological feature. These criteria have the advantage that when the clinical aspect is weaker, it has to be associated to a stronger neuroradiological finding to lead to vasculitis suspicion and vice versa.
3. Having searched among patients with diagnosed PACNS in literature, the application of a set of criteria derived from such cases, without external or internal validation, might have some methodological issues. In particular, through reverse causality, some features might seem highly specific, although they might also be present in suspected, not-confirmed PACNS cases. Criteria should have been elaborated to differentiate and identify PACNS having a control group. A case-control study comparing cases classified using such criteria with cases with brain involvement by systemic vasculitis should be in order. However, given the rarity and the diagnostic difficulties preventing diagnosis of clinicopathologically definite disease, either external or internal validation look hard at present. The only type of validation that could be done is through a Delphi method, based on an approval questionnaire circulated across the major neurovascular experts worldwide.
4. As PACNS is a rare disease, we tried to elaborate a sensitive more than a specific algorithm. This could, in some cases, lead to perform invasive investigations in patients who do not actually need them. With the increasing use of newer imaging techniques, the specificity of this tool will likely increase.

From our literature review, two aspects of the clinical picture emerged: firstly, the above described ‘focal neurological deficits’ in the absence of a clearly reported history of stroke, suggest that their onset may not always be acute. A different type of onset could be the result of a difference in the size of vessels affected, i.e. large vessel vasculitis could more easily lead to stroke-like presentation, whereas inflammation of smaller vessels could be related to less acute onsets or more diffuse symptoms, such as cognitive impairment, in some cases due to mass effect or slowly developing tissue inflammation.

Secondly, different types of visual disturbances (blurred vision, diplopia, visual field defects, scotomata) are frequently reported. It could be of interest to better investigate this aspect of the clinical presentation, to understand whether it is related to areas more vulnerable to vasculitic insults, and why.

Regarding neuroradiological features, we found that hemorrhage of any type (intraparenchymal and/or subarachnoid) is not as frequent as one would expect considering the presence of diffuse vascular damage. Some subtypes of PACNS, such as cerebral amyloid angiopathy-related inflammation (CAARI), might be more liable to hemorrhage. Differentiation of these forms would be of help to weigh the risk to benefit ratio of secondary prevention antithrombotic therapy.

The algorithm we propose should be interpreted as a preliminary first step in the PACNS diagnostic process, not as a conclusive diagnostic tool. It needs to be validated on a large cohort of patients with PACNS diagnosed according to the Calabrese and Mallek criteria [2], or better yet, only on biopsy-proven PACNS cases. If validated, it is meant to be used to early suspect the disease and to appropriately screen patients who deserve to be further studied with invasive investigations. This could help to speed diagnosis and therapy and even to prevent disability.

## References

[1] Hajj-Ali R, Furlan A, Abou-Chebel A, Calabrese L (2002). Benign angiopathy of the central nervous system: cohort of 16 patients with clinical course and long-term followup. Arthritis Rheum.

[2] Calabrese LH, Mallek JA (1988). Primary angiitis of the central nervous system. Report of 8 new cases, review of the literature, and proposal for diagnostic criteria. Medicine (Baltimore).

[3] Salvarani C, Brown RD, Calamia KT, Christianson TJH, Weigand SD, Miller DV, Giannini C, Meschia JF, Huston J, Hunder GG (2007). Primary central nervous system vasculitis: analysis of 101 patients. Ann Neurol.

[4] Twilt M, Benseler SM (2012). The spectrum of CNS vasculits in children and adults. Nat Rev Rheumatol.

[5] Volcy M, Toro ME, Uribe CS, Toro G (2004). Primary angiitis of the central nervous system: report of five biopsy-confirmed cases from Colombia. J Neurol Sci.

[6] MacLaren K, Gillespie J, Shrestha S (2005). Primary angiitis of the central nervous system: emerging variants. Q J Med.

[7] Kuker W, Gaertner S, Naegle T (2008). Vessel wall contrast enhancement: a diagnostic sign of cerebral vasculitis. Cerebrovasc Dis.

[8] Molloy ES, Singhal AB, Calabrrese LH (2008). Tumor-like mass-lesion:an under-recognised presentation of primary angiitis of the central nervous system. Ann Rheum Dis.

[9] Lee Y, Kim J, Kim E, Park SH, Yim YJ, Sohn CH, Chang KH (2009). Tumor-mimicking primary angiitis of the central nervous system: initial and follow-up MR features. Neuroradiology.

[10] Kraemer M, Berlit P (2011). Primary central nervous system vasculitis: clinical experiences with 21 new European cases. RheumatolInt.

[11] Pizzanelli C, Catarsi E, Pelliccia V, Cosottini M, Pesaresi I, Puglioli M, Moretti P, Tavoni A (2011). Primary angiitis of the central nervous system: report of eight cases from a single Italian center. J Neurol Sci.

[12] Nèel A, Auffray-Calvier E, Guillon B (2012). Challenging the diagnosis of primary angiitis of the central nervous system: a single-center retrospective study. J Rheumatol.

[13] Pfefferkorn T, Linn J, Habs M (2012). Black blood MRI in suspected large artery primary angiitis of the central nervous system. J Neuroimaging.

[14] Pourmahmoodian H, Ali GOH, Harrirchian MH (2012). Primary angiitis of the central nervous system. Acta MedicaIranica.

[15] Coronel-Restrepo N, Bonilla-Abadía F, Cortes OA (2013). Primary angiitis of the central nervous system: a report of three cases from a single colombian center. Case Rep Neurol Med.

[16] Cosottini M, Canovetti S, Pesaresi I, Desideri I, Pizzanelli C, Catarsi E, Puglioli M, Tavoni A, Bonuccelli U, Bartolozzi C (2013). 3-T magnetic resonance angiography in primary angiitis of the central nervous system. J Comput Assist Tomogr.

[17] Geri G, Saadoun D, Guillevin R, Crozier S, Lubetzki C, Mokhtari K, Amoura Z, Wechsler B, le Boutin D, Costedoat-Chalumeau N, Samson Y, Cacoub P (2014). Central nervous system angiitis: a series of 31 patients. Clin Rheumatol.

[18] Oon S, Roberts C, Gorelik A, Wicks I, Brand C (2013). Primary angiitis of the central nervous system: experience of a Victorian tertiary-referral hospital. Intern Med J.

[19] Vera-Lastra O, Sepúlveda-Delgado J, Cruz-DomínguezMdel P (2015). Primary and secondary central nervous system vasculitis: clinical manifestations, laboratory findings, neuroimaging, and treatment analysis. Clin Rheumatol.

[20] Salvarani C, Brown RD, Christianson TJ (2015). Adult primary central nervous system vasculitis treatment and course: analysis of one hundred sixty-three patients. Arthritis Rheum.

[21] Singhal AB, Topcuoglu MA, Fok JW, Kursun O, Nogueira RG, Frosch MP, Caviness VS (2016). Reversible cerebral vasoconstriction syndromes and primary angiitis of the central nervous system: clinical, imaging, and angiographic comparison. Ann Neurol.

[22] Becker J, Horn PA, Keyvani K, Metz I, Wegner C, Brück W, Heinemann FM, Schwitalla JC, Berlit P, Kraemer M (2017). Primary central nervous system vasculitis and its mimicking diseases - clinical features, outcome, comorbidities and diagnostic results - a case control study. Clin Neurol Neurosurg.

[23] De Boysson H, Boulouis G, Aouba A (2017). Adult primary angiitis of the central nervous system: isolated small-vessel vasculitis represents distinct disease pattern. Rheumatology (Oxford).

[24] De Boysson H, Zuber M, Naggara O (2014). Primary angiitis of the central nervous system. Description of the first fifty-two adults enrolled in the French cohort of patients with primary vasculitis of the central nervous system. Arthritis Rheum.

[25] Niu L, Wang L, Yin X, Li XF, Wang F (2017). Role of magnetic resonance imaging in the diagnosis of primary central nervous system angiitis. Exp Ther Med.

[26] Schuster S, Bachmann H, Thom V, Kaufmann-Buehler AK, Matschke J, Siemonsen S, Glatzel M, Fiehler J, Gerloff C, Magnus T, Thomalla G (2017). Subtypes of primary angiitis of the CNS identified by MRI patterns reflect the size of affected vessels. J Neurol Neurosurg Psychiatry.

[27] Peng LJ, Qian HR, Mao LL, Xia DY, Qi XK (2017). A clinical analysis of 5 patients with infratentorial primary angiitis of central nervous system. ZhonghuaNeiKe Za Zhi.

[28] Harsha KJ, Jagtap SA, Kapilamoorthy TR, Kesavadas C, Thomas B, Radhakrishnan N (2017). CNS small vessel vasculitis: distinct MRI features and histopathological correlation. Neurol India.

[29] Al Share B, Zakaria A, Hiner E, Iskenderian Z, Warra N (2017). Primary angiitis of the center nervous system: a clinical challenge diagnosed postmortem. Case Rep Neurol Med.

[30] Kumar PP, Rajesh A, Kandadai RM, Purohit AK, Sundaram C (2017). Primary CNS vasculitis masquerading as glioblastoma: a case report and review. Asian J Neurosurg.

[31] Takahashi K, Sato H, Hattori H, Takao M, Takahashi S, Suzuki N (2017). Case report of a 28-year-old male with the rapid progression of steroid-resistant central nervous system vasculitis diagnosed by a brain biopsy. RinshoShinkeigaku.

[32] Lee JS, Jung TY, Lee KH, Kim SK (2017). Primary central nervous system vasculitis mimicking a cortical brain tumor: a case report. Brain Tumor Res Treat.

[33] Arif S, Liaqat J, Nawaz KH, Hashmat A (2017). Focal hemispheric central nervous system vasculitis: an unusual form of primary angiitis. J Ayub Med Coll Abbottabad.

[34] Mizuno Y, Shigeto H, Yamada T, Maeda N, Suzuki SO, Kira J (2016). A case of primary central nervous system vasculitis diagnosed by second brain biopsy and treated successfully. RinshoShinkeigaku..

[35] Sun LI, Zhu L, Zhao T, Wang D, Ma D, Zhang R, Fang S (2016). A rare case of tumor-mimicking primary angiitis of the central nervous system. Mol Clin Oncol.

[36] Benson CE, Knezevic A, Lynch SC (2016). Primary central nervous system vasculitis with optic nerve involvement. J Neuroophthalmol.

[37] Pillai SH, Sreedharan SE, Menon G, Kannoth S, Pn S (2016). Primary CNS vasculitis presenting as intraventricular bleeding. Ann Indian Acad Neurol.

[38] Fang CW, Chen YC, Liao IC, Lin CC (2015). Primary granulomatous angiitis of the central nervous system with amyloid angiopathy: a case report and literature review. Neurologist..

[39] Kim S, Kim DK (2015). Psychosis in primary angiitis of the central nervous system involving bilateral thalami: a case report. Gen Hosp Psychiatry.

[40] Bajaj BK, Pandey S, Ramanujam B, Wadhwa A (2015). Primary angiitis of central nervous system: the story of a great masquerader. J Neurosci Rural Pract.

[41] Kim SI, Kim SH, Cho HJ, Kim H, Chung CK, Choi SH, Park SH (2015). Mass-forming primary angiitis of central nervous system with Rosai-Dorfmann disease-like massive histiocytosis with emperipolesis. Pathol Int.

[42] Killeen T, Jucker D, Went P, Muthurajah V, Woon K, Cesnulis E, Czaplinski A (2015). Solitary tumour-like mass lesions of the central nervous system: primary angiitis of the CNS and inflammatory pseudotumour. Clin Neurol Neurosurg.

[43] Gan C, Maingard J, Giles L, Phal PM, Tan KM (2015). Primary angiitis of the central nervous system presenting as a mass lesion. J Clin Neurosci.

[44] Huang M, Steele WJ, Baskin DS (2015). Primary central nervous system vasculitis preceded by granulomatous hypophysitis: case report with a review of the literature. Surg Neurol Int.

[45] Okunomiya T, Kageyama T, Tanaka K, Kambe D, Shinde A, Suenaga T (2014). Lymphocytic primary angiitis of the central nervous system with fan-shaped linear enhancement converging to the lateral ventricles: a case report. RinshoShinkeigaku..

[46] Safouris A, Stricker J, Michotte A, Voumvourakis K, Gazagnes MD, Tsivgoulis G (2014). Biopsy-proven fulminant primary angiitis of the central nervous system with normal arteriography: a challenging diagnosis of recurrent ischemic strokes. Neurol Sci.

[47] Michiels V, Bissay V, Michotte A (2014). Neuropsychological functioning in a patient with primary angiitis of the CNS. J Neuropsychiatr Clin Neurosci.

[48] Gaillard N, Bertrand JL, Dumitrana A, Sablot D (2014). Microaneurysms in primary angiitis of the central nervous system revealed by MRI. Cerebrovasc Dis.

[49] Rao NM, Prasad PS, Flippen CC (2014). Primary angiitis of the central nervous system presenting as unilateral optic neuritis. J Neuroophthalmol.

[50] Noh HJ, Choi JW, Kim JP, Moon GJ, Bang OY (2014). Role of high-resolution magnetic resonance imaging in the diagnosis of primary angiitis of the central nervous system. J Clin Neurol.

[51] Orr SL, Dos Santos MP, Jurencak R, Michaud J, Miller E, Doja A (2014). Central nervous system venulitis presenting as migraine. Headache..

[52] Lyra TG, Martin Mda G, Carvalho Rdo C (2013). Pseudotumoral presentation of primary central nervous system vasculitis. ArqNeuropsiquiatr..

[53] Rosenberg J, Mahta A, Koppula K, Borys E, Kesari S (2013). Cyclophosphamide responsive primary angiitis of the CNS in a 61-year-old female. Clin Neuropathol.

[54] Okeda R, Ito K, Tsumura K, Eishi Y (2013). Primary granulomatous angiitis of the CNS preferentially involving small veins with a granulomatous leukoencephalitis-like lesion in the cerebrum. Neuropathology..

[55] Yu XL, Liu AF, Ma L, Yan CZ, Zhao YY, Shan PY (2011). Primary angiitis of the central nervous system: a case report. Chin Med J.

[56] Ghavanini AA, Munoz DG (2011). Primary cerebral angiitis associated with amyloid angiopathy. Arch Neurol.

[57] Ho MG, Chai W, Vinters HV, Hathout G, Mishra S, Yim C, Valdes-Sueiras M, Nishimura R (2011). Unilateral hemispheric primary angiitis of the central nervous system. J Neurol.

[58] Tanei T, Nakahara N, Takebayashi S, Ito M, Hashizume Y, Wakabayashi T (2011). Primary angiitis of the central nervous system mimicking tumor-like lesion--case report. Neurol Med Chir (Tokyo).

[59] Pires C, Foreid H, Barroso C, Ferro JM (2011) Rapidly progressive dementia due to leukocytoclastic vasculitis of the central nervous system. BMJ Case Rep 10.1136/bcr.08.2011.4619 PMC3185387 22679329

[60] John S, Hajj-Ali RA (2014). CNS vasculitis. Semin Neurol.

[61] Carandang CG, Grant AL (2008). Delirium and isolated angiitis of the central nervous system: a case report and review. CNS Spectrums.

[62] Diamanti S, Longoni M, Agostoni EC (2019). Leading symptoms in cerebrovascular diseases: what about headache?. Neurol Sci.

[63] Mandal J, Chung SA (2017). Primary angiitis of the central nervous system. Rheum Dis Clin N Am.

[64] Beuker C, Schmidt A, Strunk D, Sporns PB, Wiendl H, Meuth SG, Minnerup J (2018). Primary angiitis of the central nervous system: diagnosis and treatment. Ther Adv Neurol Disord.

